# Sequence and phylogenetic analysis of the mitochondrial genome for the Northern Anchovy, *Engraulis mordax* (Engraulidae: Clupeiformes)

**DOI:** 10.1080/23802359.2018.1535846

**Published:** 2018-11-21

**Authors:** Olivia H. Lewis, Sean C. Lema

**Affiliations:** 1 Center for Coastal Marine Sciences, Department of Biological Sciences, California Polytechnic State University, San Luis Obispo, CA, USA

**Keywords:** mtDNA, anchovies, mitochondrion, fishery, omega-3, California current

## Abstract

The northern anchovy, *Engraulis mordax*, is a small planktivorous fish from the northeastern Pacific Ocean that is an important forage for fishes, birds, and marine mammals, and is also the target of a commercial fishery. Here, we assembled a complete 16,664 bp genome for the *E. mordax* mitochondrion, which encodes for *12S* and *16S* rRNAs, 22 tRNAs, 13 protein-coding genes, and a 1016 bp D-loop in the characteristic arrangement of Order Clupeiformes. Phylogenetic analysis confirmed the evolutionary relatedness of *E. mordax* to other fishes of Family Engraulidae within Order Clupeiformes, but also indicated non-monophyly for the herring family, Clupeidae.

The northern anchovy (*Engraulis mordax* Girard 1854) is a pelagic schooling fish found in coastal waters of the California Current ecosystem from British Columbia, Canada, to the Gulf of California, Mexico. Northern anchovies feed on copepods, euphausiids, and meroplankton larvae by filtering with gill rakers or by picking larger, individual prey (van der Lingen et al. 2009; Chiappa-Carrara and Gallardo-Cabello 1993), and are themselves forage for larger fishes, seabirds, and marine mammals.

Anchovies, sardines, and herrings of Order Clupeiformes comprised 13.4% of global fisheries in 2013–2014 (FAO 2016). Commercial catches of northern anchovy, however, have varied widely (Checkley and Barth 2009), as northern anchovy populations fluctuate with changing oceanographic conditions (Lluch-Belda et al. 1992). From 1952 to 1957, catches of *E. mordax* averaged >28,000 tons a year – but declined to <3,100 tons from 1958 to 1965 – only to increase to >73,000 tons between 1966 and 1975 (Huppert et al. 1980). Despite a catch limit of 25,000 tons, landings averaged 8,242 tons between 2001 and 2015 (CDFW 2016). These recent low landings are in part due to a collapse of *E. mordax* between 2009 and 2011 (MacCall et al. 2016).

In this study, we report the first complete mitochondrial DNA genome sequence for the northern anchovy. The northern anchovy has been divided into three populations based on evidence from meristic characters and electrophoretic protein variation (McHugh 1951; Vrooman and Palmoa 1975). Here, a male *E. mordax* (123.8 mm standard length, 16.72 g mass) from the ‘central population’ was collected on 10 July 2017 from the pier in Cayucos, CA, USA (35.447172 N, 120.907368 W). Tissue from this specimen was archived in the Natural History Museum of Los Angeles County (Tissue #T-001272).

DNA was extracted from skeletal muscle (DNeasy Blood and Tissue Kit; Qiagen, Valencia, CA) and amplified as overlapping PCR products (GoTaq^®^ Long PCR Master Mix, Promega Corp., Madison, WI) using primers designed to partial sequences from *E. mordax* for *16S* (GenBank accession nos. AB246182, DQ912072) and 12S (LC091575, LC091576, DQ912037), cytochrome oxidase I (JQ398444, FJ164580), cytochrome b (AY923775, JQ012421), and the D-loop (GU136672, GU136673). Resulting PCR products were Sanger sequenced (Molecular Cloning Laboratory, South San Francisco, CA), assembled (Sequencher v5; Gene Codes Corp., Ann Arbor, MI), and annotated using MitoFish (Iwasaki et al. 2013).

The complete mitochondrion genome of *E. mordax* (MH613715) was 16,664 bps with 13 protein-coding genes, 2 rRNAs, 22 tRNAs, and D-loop region of 1016 bp, located in the arrangement typical of other clupeiform fishes. Phylogenetic analysis (Figure 1) confirmed that *E. mordax* is closely related to other “New World clade” anchovies (Lavoué et al. 2017a) – including the Japanese anchovy (*Engraulis japonicus*) and European anchovy (*Engraulis encrasicolus*) – within monophyletic Subfamily Engraulinae of Family Engraulidae, which also contains monophyletic Subfamily Coiliinae. All protein-coding genes showed >95% amino acid identity to homologous proteins from *E. japonicus* and *E. encrasicolus* – except *ATPase8*, which showed 89–90% identity. Interestingly, phylogenetic analysis using complete mitogenomes indicated that Family Clupeidae is not monophyletic, and the taxonomic status of Family Chirocentridae (wolf herrings) should be further evaluated (see, Lavoué et al. 2017b).

**Figure 1.**
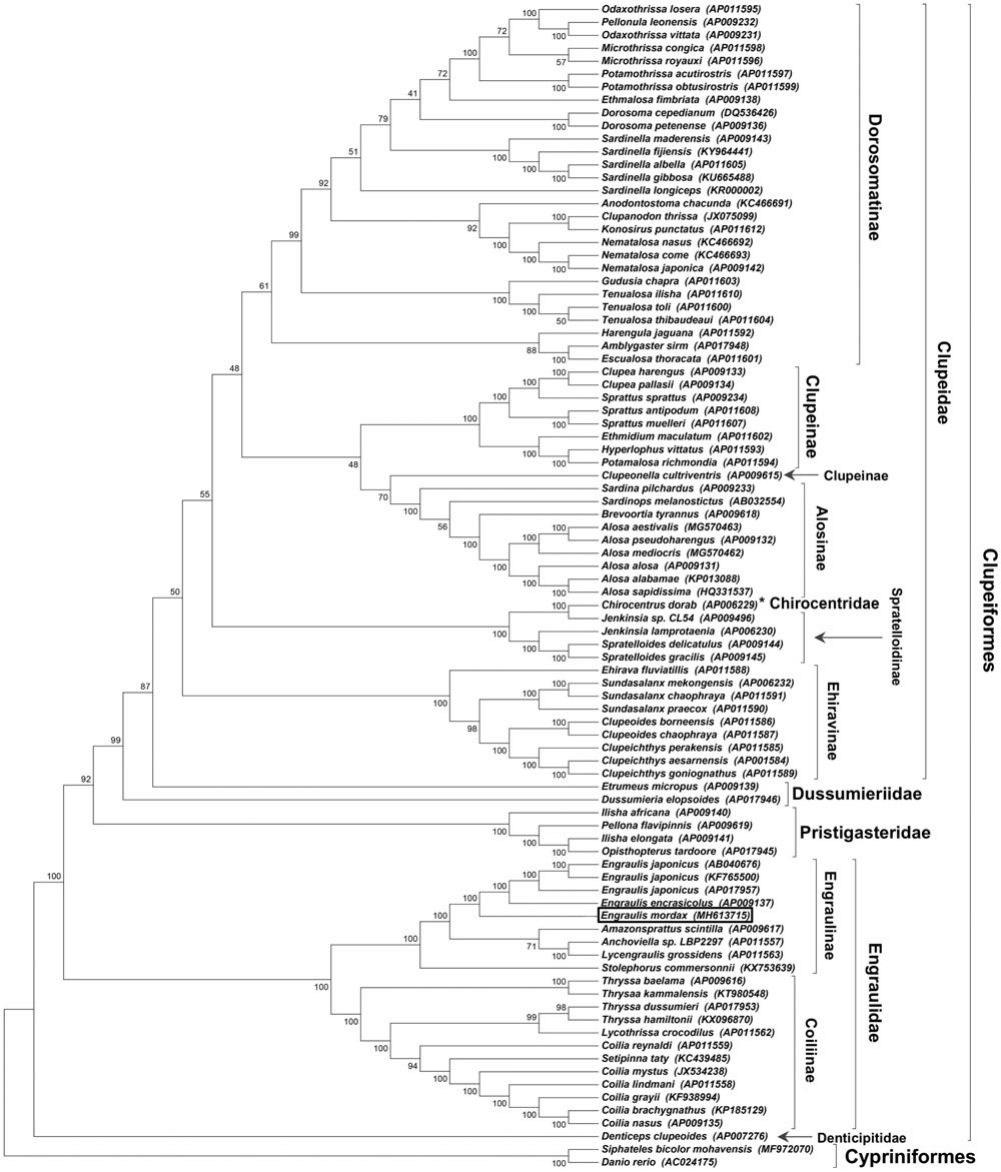
Consensus maximum-likelihood phylogenetic tree of the complete mitogenome of *E. mordax* (indicated as boxed) (GenBank accession no. MH613715) and other fishes of Order Clupeiformes. Phylogenetic analysis confirms that *E. mordax* belongs to the clade of anchovies of Subfamily Engraulinae (Family Engraulidae), which also contains fishes of the monophyletic Coiliinae subfamily. Phylogenetic analysis also indicated Family Clupeidae is paraphyletic due to its relationship with Family Chirocentridae (see asterisk; represented here by *Chirocentrus dorab*, AP006229), confirming recent findings by Lavoué and coworkers (2017b). Complete nucleotide sequences for each species were aligned using Clustal X software (Larkin et al. 2007), and the tree was constructed using all nucleotide sites with a maximum likelihood model and pairwise gap deletion using MEGA v7 (Kumar et al. 2016). The tree was rooted using complete mitogenomes of two fishes from Order Cyprinifomes: the Mohave Tui Chub, *Siphateles bicolor mohavensis* (Glaser et al. 2017), and Zebrafish, *Danio rerio.* Bootstap values (1000 replicates) are shown for each node, and GenBank accession nos. are provide in parentheses accompanying each taxon.
